# Jenner Monument

**Published:** 1852-02

**Authors:** 


*Monument to Dr. Jenner.* — An association of distinguished gentlemen in London, have undertaken the commendable enterprise of erecting a bronze statue of Jenner in some public situation in London, as a fitting testimonial of respect for the memory of the author of one of the grandest discoveries ever achieved by human genius, and one which has conferred inestimable benefits on the human race. Entertaining the belief that the medical profession in other countries will esteem it a privilege to contribute toward this object, the invitation is extended to American physicians. Individual contributions thus far procured in this country have generally been limited to *one dollar*. Persons desirous of participating in this good work may transmit their donations to Prof. Charles A. Lee, one of the authorized agents for the fund in this country, or to Prof. Robley Dunglison, of Philadelphia.

